# Impacts of commingling cattle from different sources on their physiological, health, and performance responses during feedlot receiving

**DOI:** 10.1093/tas/txaa204

**Published:** 2020-11-10

**Authors:** Jacob B Wiegand, Reinaldo F Cooke, Alice P Brandão, Kelsey M Schubach, Eduardo A Colombo, Courtney L Daigle, Glenn C Duff, Vinicius N Gouvêa

**Affiliations:** 1 Department of Animal Science − Texas A&M University, College Station, TX; 2 Prairie Research Unit − Mississippi State University, Prairie, MS; 3 Clayton Livestock Research Center − New Mexico State University, Clayton, NM

**Keywords:** commingling, performance, physiology, receiving cattle, respiratory disease, stress

## Abstract

This experiment compared physiological, health, and performance responses of beef heifers assigned to different commingling schemes (one, two, or four sources per pen) during a 56-d feedlot receiving period. Ninety-six recently weaned Angus-influenced heifers were obtained from an auction facility. Heifers originated from four cow-calf ranches, and were reared in the same herd within each ranch since birth. Heifers were loaded into two livestock trailers at the auction yard (two sources per trailer; d −2), arranged in two sections of each trailer according to source, and transported for 10 h to stimulate the stress of a long-haul. Heifers were not mixed with cohorts from other sources prior to and at the auction yard. Upon arrival (d −2), shrunk body weight (BW) was recorded and heifers were maintained in four paddocks by source with ad libitum access to a complete starter feed and water for 36 h. On d 0, heifers were ranked by source and shrunk BW and allocated to 1 of 24 drylot pens (four heifers per pen) containing: 1) heifers from a single source (1SRC, *n* = 8), 2) heifers from two sources (2SRC, *n* = 8), or 3) heifers from four sources (4SRC, *n* = 8). From d 0 to d 55, heifers had free-choice access to the complete starter feed and water. Heifers were assessed daily for symptoms of bovine respiratory disease (BRD), and feed intake was recorded from each pen daily. Blood samples were collected on d 0, d 6, d 13, d 27, d 41, and d 55, and shrunk BW (after 16 h of water and feed withdrawal) was recorded on d 56 for average daily gain (ADG). No treatment differences were noted (*P* ≥ 0.56) for heifer ADG (mean ± SE = 0.853 ± 0.043 kg/d), final shrunk BW, feed intake, and feed efficiency. No treatment differences were noted (*P* ≥ 0.27) for plasma concentrations of cortisol and haptoglobin, and serum concentrations of antibodies against BRD viruses and *Mannheimia haemolytica*. No treatment differences were noted (*P* ≥ 0.17) for incidence of BRD (mean ± SE = 59.3 ± 5.0%) or mortality. The proportion of heifers diagnosed with BRD that required three antimicrobial treatments to regain health increased linearly (*P* = 0.03) according to the number of sources (0.0, 12.3, and 20.8% of 1SRC, 2SRC, and 4SRC heifers, respectively; SEM = 7.0). Hence, commingling heifers from different sources did not impact performance, physiological responses, and BRD incidence during a 56-d receiving period, although recurrence of BRD after the second antimicrobial treatment increased according to commingling level.

## INTRODUCTION

Feedlot receiving is one of the most challenging phases within the beef production cycle, when cattle are exposed to a multitude of stressors that impair their immunocompetence and growth (Duff and Galyean, 2007). Commingling is recognized as a critical stressor during feedlot receiving, and typically occurs shortly after major stressful events such as weaning and road transport (Cooke, 2017). When cattle from various sources are commingled in the same pen, social hierarchy is destabilized and psychological stress reactions are provoked until social structure is re-established (Loerch and Fluharty, 1999). Commingling can be perceived by cattle as an acute or chronic stressor depending upon how much time is required for social structures to reform and stabilize (Grant and Albright, 2001).

Several epidemiological studies recognized commingling as a risk factor for bovine respiratory disease (BRD) in feedlots (Taylor et al., 2010). Nonetheless, few experimental research trials have attempted to examine the magnitude of commingling-induced stress and its consequences to immunocompetence and productivity of receiving cattle. Step et al. (2008) reported reduced performance and increased BRD incidence in receiving pens containing steers from multiple sources compared to pens with single source steers. Ribble et al. (1998) surveyed receiving yards that commingled receiving cattle, and reported that pens with fewer cattle sources had reduced BRD incidence compared with pens with cattle from a larger number of sources. These research efforts, however, did not quantify number of cattle sources in commingled pens nor evaluated stress and physiological responses to commingling.

To our knowledge, no experimental research has investigated if number of cattle sources being commingled impact resultant stress, immune, and productive responses of receiving cattle. We hypothesized that commingling will elicit stress responses that influence cattle immunocompetence and growth, and such outcomes intensify according to the number of cattle sources mixed within the receiving pen. Therefore, this experiment compared physiological, health, and performance responses of beef heifers assigned to different commingling schemes (one, two, or four sources pen pen) during a 56-d feedlot receiving period.

## MATERIALS AND METHODS

This experiment was conducted at the New Mexico State University − Clayton Livestock Research Center (Clayton, NM). All animals were cared for in accordance with acceptable practices and experimental protocols reviewed and approved by the New Mexico State University − Institutional Animal Care and Use Committee (#2018-028).

### Animals and Treatments

Ninety-six recently weaned Angus-influenced heifers were obtained from a commercial auction facility (Cattlemen’s Livestock Commission Company, Dalhart, TX) and utilized in this experiment. Heifers originated from four cow-calf ranches and were reared in the same herd within each ranch since birth. Besides origin, no additional heifer management history was available. Heifers were not mixed with cohorts from other sources prior to and at the auction yard. On the day of purchase (d −2; 0800 hours), heifers were loaded into two commercial livestock trailers (Legend 50’ cattle liner; Barrett LLC, Purcell, OK), arranged in two sections of each trailer according to source (being two sources per trailer), and transported for 700 km (10 h) to stimulate the stress of a long-haul (Cooke, 2017). On d −2 (1800 hours), heifers were unloaded and immediately weighed (initial shrunk body weight (BW) = 239 ± 2 kg), and maintained in four paddocks according to source with ad libitum access to water and a complete starter feed (RAMP; Cargill Corn Milling, Blair, NE; Schneider et al., 2017) for a 36-h rest period.

**Figure 1.**
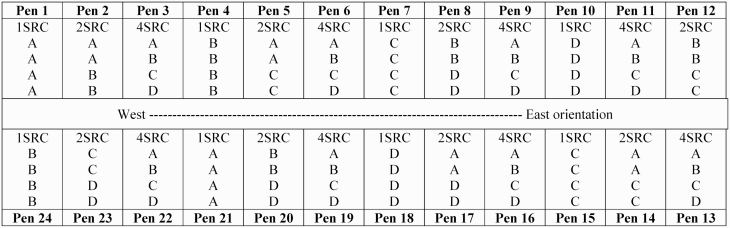
Arrangement of heifers and pens according to cow-calf sources (A, B, C, or D) and treatments (1SRC = 1 source; 2SRC = 2 sources; 4SRC = 4 sources). Each pen contained four heifers, and each treatment contained eight pens.

On d 0 of the experiment, heifers were ranked by source and initial shrunk BW and allocated to 1 of 24 drylot pens (10 × 5 m; four heifers per pen) containing: 1) heifers from a single source (1SRC, *n* = 8), 2) heifers from two sources (2SRC, *n* = 8), or 3) heifers from four sources (4SRC, *n* = 8). Heifers were assigned to pens in a manner that initial shrunk BW was equivalent across pens and treatments, following the design illustrated in Figure 1. A solid construction tarp was placed on the sides of all pens, on top of the original metal pipe fencing, to minimize interaction of heifers from differing pens. All tarps were firmly secured using industrial-strength nylon ties to prevent tarp movement that would influence heifer behavior.

On d 0 of the experiment, heifers were vaccinated against *Clostridium* (Covexin 8; Merck Animal Health, Madison, NJ), *Mannheimia haemolytica*, *bovine respiratory syncytial virus*, *bovine herpesvirus-1*, *bovine viral diarrhea virus 1 and 2*, and *parainfluenza-3 virus* (Vista Once SQ; Merck Animal Health), administered an anthelmintic (Safe-Guard, Merck Animal Health), and received a growth-promoting implant (Synovex-H; Zoetis, Florham Park, NJ). Heifers had free-choice access to water and the aforementioned starter feed (RAMP; Cargill Corn Milling) from d 0 to d 55, which was fed once daily (0800 hours) in a manner to yield 10% residual orts (Colombo et al., 2019).

### Sampling

Samples of starter feed were collected weekly, pooled across weeks, and analyzed for nutrient content (Dairy One Forage Laboratory, Ithaca, NY). Feed intake (dry matter basis) was evaluated from d 0 to 55 from each pen by collecting and weighing offered and nonconsumed feed daily. Samples of offered and nonconsumed feed were dried for 96 h at 50 °C in forced-air ovens for dry matter calculation. Feed intake of each pen was divided by the number of heifers within each pen, and expressed as kg per heifer/day. Heifer shrunk BW was recorded again on d 56, after 16 h of water and feed withdrawal. Shrunk BW values from d −2 and 56 were used to calculate heifer average daily gain (ADG) during the experiment. Total BW gain and feed intake of each pen were used for feed efficiency calculation. Blood samples were collected from all heifers on d 0, d 6, d 13, d 27, d 41, and d 55 into commercial blood collection tubes (Vacutainer, 10 mL; Becton Dickinson, Franklin Lakes, NJ) containing either no additive or freeze-dried sodium heparin for serum and plasma collection, respectively. Hair samples were collected from the tail switch on d 0, d 13, d 27, d 41, and d 55 as in Schubach et al. (2017).

Heifers were observed daily for symptoms of BRD according to the DART system (Zoetis) and Sousa et al. (2019), using rectal temperature ≥40.0 °C (GLM-500, GLA Agricultural Electronics, San Luis Obispo, CA) as clinical criterion, and received antimicrobial treatment similar to Lopez et al. (2018). More specifically, heifers diagnosed with BRD received florfenicol with flunixin meglumine (Resflor Gold, Merck Animal Health) at 1 mL/7.6 kg of BW subcutaneously as the first antimicrobial administered, followed by a 5-d moratorium. Heifers diagnosed with BRD after first antimicrobial treatment were administered ceftiofur crystalline free acid (Excede; Zoetis) at 1 mL/30.3 kg of BW, followed by another 5-d moratorium. Heifers diagnosed with BRD after the second antimicrobial treatment were administered oxytetracycline (Bio-Mycin 200; Boehringer Ingelheim, Ridgefield, CT) at 1 mL/10 kg of BW. Heifers diagnosed with BRD after the third antimicrobial treatment would be removed from the experiment; however, none of the heifers were diagnosed with BRD after the third treatment. Heifer mortality was observed daily.

### Laboratorial Analyses

Feed samples were analyzed by wet chemistry procedures for concentrations of crude protein (method 984.13; AOAC, 2006), acid detergent fiber (method 973.18 modified for use in an Ankom 200 fiber analyzer, Ankom Technology Corp., Fairport, NY; AOAC, 2006), and neutral detergent fiber using a-amylase and sodium sulfite (Van Soest et al., 1991; modified for use in an Ankom 200 fiber analyzer, Ankom Technology Corp.). Net energy for maintenance and gain were calculated using the equations proposed by NRC (2000). Nutrient profile of the starter feed was (dry matter basis) 22.1% crude protein, 38.3% neutral detergent fiber, 19.1% acid detergent fiber, 1.83 Mcal/kg of net energy for maintenance, and 1.20 Mcal/kg of net energy for gain.

After collection, all blood samples were placed immediately on ice, centrifuged (2,500 × g for 30 min; 4 °C) for plasma or serum harvest, and stored at −80 °C on the same day of collection. All plasma samples were analyzed for concentrations of cortisol (radioimmunoassay kit #07221106, MP Biomedicals, Santa Ana, CA; Colombo et al., 2019) and haptoglobin (Cooke and Arthington, 2013). Serum samples collected on d 0, d 13, d 27, d 41, and d 55 were analyzed for antibodies against *bovine respiratory syncytial virus* (#P00651-2; IDEXX Switzerland AG, Liebefeld-Bern, Switerland), *bovine herpesvirus-1* (#99-41459; IDEXX), *parainfluenza-3 virus* (#P0652-2; IDEXX), *bovine viral diarrhea viruses* types I and II (#99-44000; IDEXX), and *Mannheimia haemolytica* (BIOK139 Monoscreen AbELISA; Bio-X Diagnostics S.A., Rochefort, Belgium). Only samples from heifers not diagnosed with BRD were analyzed for antibodies against BRD pathogens to ensure that this response was associated with vaccine efficacy rather than pathogenic infection (Callan, 2001). The intra- and interassay CV were, respectively, 5.5% and 6.8% for haptoglobin, 5.8% and 4.6% for cortisol, 3.1% and 5.7% for *bovine respiratory syncytial virus*, 1.0% and 4.1% for *bovine respiratory syncytial virus*, 3.6% and 3.5% for *bovine herpesvirus-1*, 2.8% and 4.9% for *bovine viral diarrhea viruses*, and 1.6% and 2.4% for *M. haemolytica*. Hair samples were analyzed for cortisol concentrations as in Schubach et al. (2017), with an intra- and interassay CV of 3.7% and 6.1%, respectively.

### Statistical Analysis

All data were analyzed using pen as the experimental unit, and Satterthwaite approximation to determine the denominator degrees of freedom for tests of fixed effects. Quantitative data were analyzed using the MIXED procedure of SAS (SAS Inst. Inc., Cary, NC), whereas binary data were analyzed using the GLIMMIX procedure of SAS (SAS Inst. Inc.). All models included heifer source as independent fixed variable in addition to pen(treatment) and heifer(pen) as random variables, but for feed intake and efficiency that used pen(treatment) as random variable without heifer source as fixed variable. Model statements for BW parameters, feed efficiency, and morbidity-related results contained the effects of treatment. Model statements for feed intake, cumulative BRD incidence, blood and hair variables contained the effects of treatment, day, and the resultant interaction. Plasma, serum, and hair variables were analyzed using results from d 0 as independent covariate. The specified term for all repeated statements was day, with pen(treatment) as subject for feed intake and efficiency, and heifer(pen) as subject for all other analyses. The covariance structure used was first-order autoregressive, which provided the smallest Akaike information criterion and hence the best fit for all variables analyzed. All results are reported as least square means, or covariately adjusted least square means for blood and hair variables. Orthogonal contrasts were tested to determine if number heifer sources within a pen yielded linear or quadratic responses. Significance was set at *P* ≤ 0.05 and tendencies were determined if *P* > 0.05 and ≤ 0.10. Repeated measures are reported according to main treatment effects if the treatment × day interaction was *P* > 0.10.

## RESULTS

As designed, initial shrunk BW (d −2) did not differ (*P* ≥ 0.98) among treatments (Table 1). Average daily gain and final BW also did not differ (*P* ≥ 0.60) among treatments (Table 1). No treatment differences were noted (*P* ≥ 0.56) for feed intake and feed efficiency during the experiment (Table 1).

**Table 1.** Performance parameters of beef heifers commingled (2SRC = two sources; *n* = 8; 4SRC = four sources, *n* = 8) or not (1SRC = single source, *n* = 8) with cohorts from different cow-calf sources during a 56-d feedlot receiving period^*a*^

					Contrasts (*P*-value)^*b*^	
Item	1SRC	2SRC	4SRC	SEM	Linear	Quadratic
Initial body weight, kg	240	239	240	7	0.98	0.97
Final body weight, kg	287	290	286	7	0.85	0.72
Average daily gain, kg/d	0.849	0.887	0.813	0.076	0.66	0.60
Feed intake (kg/day)	6.48	6.55	6.32	0.23	0.56	0.67
Feed efficiency (g/kg)	132	136	127	9	0.66	0.67

^*a*^ Heifer shrunk body weight was recorded on d −2 (initial; after 10-h road transport) and d 56 (final; after 16 h of water and feed withdrawal), and used for average daily gain calculation. Heifers received a complete starter feed (RAMP; Cargill Corn Milling, Blair, NE) for ad libitum consumption from d 0 to d 55. Feed intake was recorded daily measuring offer and refusals from each pen, divided by the number of heifers within each pen, and expressed as kg per heifer/d. Feed efficiency was calculated using total body weight gain (in grams), and total feed intake (kg of dry matter) of each pen during the experimental period.

^*b*^ Orthogonal contrasts were tested to determine if number of cow-calf sources within a pen affected performance responses linearly or quadratically.

No treatment differences were detected (*P* ≥ 0.68) for concentrations of plasma cortisol, plasma haptoglobin, and hair cortisol (Table 2), whereas day effects were detected (*P* < 0.01) for all these variables (Table 3). No treatment differences were detected (*P* ≥ 0.27) for serum antibodies against BRD viruses and *M. haemolytica* (Table 2), which increased (day effects; *P* < 0.01) across treatments with the advance of the experiment (Table 3).

**Table 2.** Physiological responses from beef heifers commingled (2SRC = two sources; *n* = 8; 4SRC = four sources, *n* = 8) or not (1SRC = single source, *n* = 8) with cohorts from different cow-calf sources during a 56-d feedlot receiving period^a,b^

					Contrasts (*P*-value)^*c*^	
Item	1SRC	2SRC	4SRC	SEM	Linear	Quadratic
Hormones and metabolites						
Plasma cortisol, ng/mL	22.0	21.1	21.3	1.7	0.82	0.75
Plasma haptoglobin, mg/mL	0.848	0.844	0.896	0.089	0.68	0.86
Hair cortisol, pg/mg of hair	3.85	3.80	3.84	0.18	0.99	0.82
Serum antibodies against respiratory viruses						
*Parainfluenza-3 virus*	73.4	63.7	63.0	8.7	0.34	0.46
*Bovine respiratory syncytial virus*	84.2	81.7	80.7	16.5	0.88	0.94
*Bovine viral diarrhea viruses type I and II*	51.4	74.1	79.9	14.1	0.21	0.40
*Bovine herpesvirus-1*	182	175	195	22	0.59	0.63
*Maenhemia haemolytica*	56.1	49.7	65.3	8.26	0.28	0.33

^*a*^ Blood samples were collected on d 0, d 6, d 13, d 27, d 41, and d 55. Hair samples were collected on d 0, d 13, d 27, d 41, and d 56 as in Schubach et al. (2017). Results from d 0 were used as covariate in each respective analysis.

^*b*^ Heifers received vaccination against respiratory pathogens on d 0 (Vista Once SQ; Merck Animal Health, Madison, NJ). Samples collected on d 0, d 13, d 27, d 41, and d 55 were analyzed and results expressed as sample:positive control ratio (%) as in Cooke et al. (2020). Results from d 0 was used as covariate in each respective analysis.

^*c*^ Orthogonal contrasts were tested to determine if number of cow-calf sources within a pen affected performance responses linearly or quadratically.

**Table 3.** Serum concentrations of antibodies against *parainfluenza-3 virus* (PI3), *bovine respiratory syncytial virus* (BRSV), *bovine viral diarrhea viruses types I and II* (BVD-1), *bovine herpesvirus-1* (BHV), and *Maenhemia haemolytica* (MH), plasma concentrations of cortisol (ng/mL) and haptoglobin (mg/dL), and concentrations of cortisol in tail-switch hair (HC, pg/mg of hair) from beef heifers during a 56-d feedlot receiving period^*^

	Serum antibodies against respiratory pathogens					Hormones and metabolites		
Day	PI3	BRSV	BVDV	BHV	MH	Cortisol	Haptoglobin	HC
0	27.6^c^	36.5^b^	20.6^d^	92.0^b^	30.3^c^	19.4^d^	0.878^c^	3.51^b^
6	—	—	—	—	—	9.22^f^	1.57^a^	—
13	41.7^b^	63.6^a^	34.8^d^	123^b^	46.2^b^	14.5^e^	1.06^b^	3.75^b^
27	69.5^a^	79.6^a^	52.9^c^	192^a^	47.7^b^	24.9^c^	0.736^cd^	3.62^b^
41	70.3^a^	75.4^a^	82.5^b^	200^a^	61.5^a^	28.1^b^	0.636^d^	4.48^a^
55	67.6^a^	76.9^a^	101.6^a^	204^a^	64.8^a^	31.0^a^	0.330^e^	3.47^b^
SEM	5.9	10.3	9.0	14	6.8	1.47	0.075	0.18
*P-value*	< 0.01	< 0.01	< 0.01	< 0.01	< 0.01	< 0.01	< 0.01	< 0.01

^*^ Within columns, values with different superscripts differ (*P* ≤ 0.05). Serum antibodies results expressed as sample:positive control ratio (%) as in Cooke et al. (2020). Heifers received vaccination against respiratory pathogens on d 0 (Vista Once SQ; Merck Animal Health, Madison, NJ).

**Figure 2.**
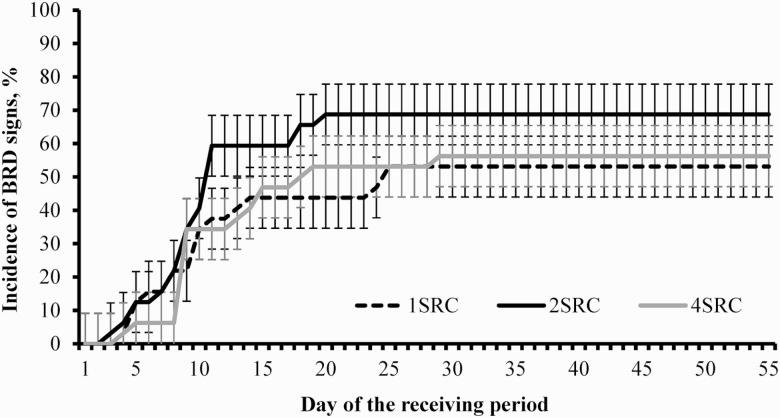
Cumulative incidence of bovine respiratory disease (BRD) symptoms beef heifers commingled (2SRC = two sources; *n* = 8; 4SRC = four sources, *n* = 8) or not (1SRC = single source, *n* = 8) with cohorts from different cow-calf sources during a 56-d feedlot receiving period. Heifers were observed daily for symptoms of BRD according to the DART system (Zoetis, Florham Park, NJ) and Sousa et al. (2019), and received antimicrobial treatment as described in Lopez et al. (2018). No treatment differences nor the treatment × day interaction were detected (*P* ≥ 0.24).

No treatment differences were detected (*P* ≥ 0.24) for incidence of BRD (Table 4; Figure 2). Within heifers diagnosed with BRD during the experiment, no treatment differences were noted (*P* ≥ 0.12) in the proportion of heifers that required one or two antimicrobial treatments to regain health (Table 4). However, the proportion of heifers that required three antimicrobial treatments to regain health increased linearly (*P* = 0.03) according to the number of sources within the receiving pen (Table 4). No treatment differences were detected (*P* ≥ 0.17) for mortality rate during the experiment (Table 4).

**Table 4.** Health responses from beef heifers commingled (2SRC = two sources; *n* = 8; 4SRC = four sources, *n* = 8) or not (1SRC = single source, *n* = 8) with cohorts from different cow-calf sources during a 56-d feedlot receiving period^*a*^

					Contrasts (*P*-value)^*b*^	
Item	1SRC	2SRC	4SRC	SEM	Linear	Quadratic
Heifers treated for respiratory disease, %	53.1	68.7	56.2	9.7	0.99	0.24
One treatment required	73.8	66.9	70.6	11.0	0.90	0.66
Two treatment required	31.7	20.9	8.88	9.89	0.12	0.78
Three treatments required	0.00	12.3	20.8	7.0	0.03	0.32
Mortality, %	9.37	0.00	3.12	3.54	0.35	0.17

^*a*^ Heifers were observed daily for symptoms of BRD according to the DART system (Zoetis, Florham Park, NJ), and received antimicrobial treatment as in Lopez et al. (2018).

^*b*^ Orthogonal contrasts were tested to determine if number of cow-calf sources within a pen affected performance responses linearly or quadratically.

## DISCUSSION

The heifers used in this experiment were considered high-risk as their management and health history were not fully known (Wilson et al., 2017; Sousa et al., 2019). All heifers were exposed to the stress of transport, initial processing, and exposure to a new environment within a 48-h period, whereas the combination of these stressors impact physiological and immune responses in cattle (Duff and Galyean, 2007; Cooke, 2017). The day effects noted for cortisol (plasma and hair) and haptoglobin concentrations across treatments corroborate that heifers experienced the adrenocortical and acute phase protein reactions provoked by road transport and feedlot entry (Cooke et al., 2013; Lippolis et al., 2017). These stress-induced physiological and inflammatory responses impair cattle immunity, corroborating the substantial incidence of BRD observed in this experiment (Cooke, 2017). Therefore, this experimental model represented the stress and health challenges typically experienced by high-risk cattle during feedlot receiving (Duff and Galyean, 2007).

Commingling is one of the main stressors experienced by receiving cattle, and considered a major predisposing cause for BRD in feedlots (Loerch and Fluharty, 1999; Taylor et al., 2010). Step et al. (2008) reported that average daily gain was reduced (1.25 vs. 1.34 kg/d) and BRD incidence was increased (41.9% vs. 11.1%) during a 42-d receiving period in pens containing steers from multiple sources compared to pens containing steers that originated from a single source. However, these authors acknowledged several shortcomings in their research design, including the contribution of previous steer management and genetic potential to research outcomes, different transportation distances, and an unknown number of cattle sources used in the multiple-sourced pens. Arthington et al. (2003) compared performance and acute-phase protein responses of single-sourced newly-weaned calves assigned to a 2 × 2 factorial design, including road-transport and commingling with auction-originated calves as main factors. Commingling did not impact acute-phase and performance responses, but authors implied that previous management of auction-originated calves may have biased the commingling treatment. Ribble et al. (1998) reported a positive association between BRD incidence and number of cattle sources within receiving pens, but without quantifying number of sources nor evaluating cattle performance traits. Based on these gaps in knowledge, this experiment was designed to examine productive, physiological and immunological implications of commingling, while exploring different levels of commingling and balancing experimental treatments according to cattle source.

We hypothesized that commingling would heighten the cortisol and haptoglobin responses that cattle experience during feedlot receiving, which in turn would reduce performance responses and increase BRD incidence. Both adrenocortical and acute-phase protein reactions are known to impair productive responses and immunocompetence in cattle, particularly when these reactions are elicited by stressors (Berry et al., 2004; Carroll and Forsberg, 2007; Cooke, 2017). However, heifer performance, physiological responses, and overall BRD incidence were not affected by commingling during the 56-d receiving period. Acquired immunity against BRD pathogens were also not impacted by commingling, as serum concentrations of antibodies against these antigens increased similarly across treatments during the 56-d receiving period (Richeson et al., 2008). These findings corroborate Arthington et al. (2003), although these authors did not report morbidity or BRD incidence in their study. Nevertheless, all the 1SRC heifers diagnosed with BRD regained health without the need for a third treatment, whereas this response increased linearly according to number of heifer sources within the receiving pen. Number of antimicrobial treatments to BRD is often associated negatively with ADG and feed efficiency in receiving cattle (Thompson et al., 2012; Blakebrough-Hall et al., 2020). These latter outcomes partially support our hypothesis as level of commingling impacted heifer competence to recover from BRD upon antimicrobial treatments, but without any benefits to heifer performance nor changes in the physiological responses measured herein.

Collectively, this experiment did not observe major negative impacts of commingling on performance and health responses of feedlot heifers during a 56-d receiving period. This is the first research investigating different levels of commingling and accounting for cattle source. To achieve our objectives, heifers were originated from four cow-calf ranches and housed in pens with four heifers, in a manner that commingling treatments and pens were balanced for heifer source. Either one or two heifers from the same source (4SCR and 2SCR, respectively) were housed together in commingled pens. Cattle are social animals and may form group sizes containing 20 to 100 individuals in free-living populations (Bouissou et al., 2001), whereas young cattle may form subgroups up to 25 individuals (Rankine and Donaldson, 1968; Sato et al., 1987). Cattle also form intricate smaller social groups within the herd, and grouping cattle according to the ranch of origin may result in either conservation or disruption of social hierarchy (Hagen and Broom, 2003). Epidemiological studies reporting increased BRD in commingled cattle surveyed feedlots with large pen sizes (i.e., ≥50 animals per pen; Alexander et al., 1989; Ribble et al., 1998), whereas Step et al. (2008) evaluated commingling effects in receiving pens with 15 calves per pen. Therefore, the lack of substantial commingling effects noted herein can be associated with the number of heifers from the same source assigned to 2SRC and 4SRC pens, which may have limited the occurrence and subsequent disruption of pre-existing social groups during feedlot receiving.

In conclusion, commingling heifers from two or four different cow-calf sources did not impact performance and overall BRD incidence during a 56-d receiving period, despite increasing the need for antimicrobial treatments for BRD. Perhaps the number of heifers assigned to commingled pens, and resultant pre-existing social groups, was not sufficient to provoke major stress reactions from social disruption. Group size is directly associated with the time required for social stabilization, given that cattle have difficulty remembering large numbers of individuals and their social status to establish hierarchy (Kondo and Hurnik, 1990; Rind and Phillips, 1999). Therefore, experimental research to further explore the impacts of commingling receiving cattle are warranted, particularly designs using large pen and groups sizes typical of commercial feedyards (Alexander et al., 1989; Ribble et al., 1998).
